# Advanced Oxidation Protein Products and Carbonylated Proteins Levels in Endovascular and Open Repair of an Abdominal Aortic Aneurysm: The Effect of Pre-, Intra-, and Postoperative Treatment

**DOI:** 10.1155/2019/7976043

**Published:** 2019-05-08

**Authors:** Bogna Gryszczyńska, Magdalena Budzyń, Dorota Formanowicz, Piotr Formanowicz, Zbigniew Krasiński, Natalia Majewska, Maria Iskra, Magdalena P. Kasprzak

**Affiliations:** ^1^Department of General Chemistry, Poznan University of Medical Sciences, Poznań, Poland; ^2^Department of Clinical Biochemistry and Laboratory Medicine, Poznan University of Medical Sciences, Poznań, Poland; ^3^Institute of Computing Science, Poznan University of Technology, Poznań, Poland; ^4^Institute of Bioorganic Chemistry, Polish Academy of Sciences, Poznań, Poland; ^5^Department of General and Vascular Surgery, Poznan University of Medical Sciences, Poznań, Poland; ^6^Department of Clinical Radiology, Poznan University of Medical Sciences, Poznań, Poland

## Abstract

**Background:**

In recent years, a rapid increase in studies focusing on the role of oxidative stress in the pathogenesis of an abdominal aortic aneurysm (AAA) has been observed. Oxidative modifications of proteins are infrequently evaluated in reference to AAA.

**Objectives:**

The intensity of oxidative protein modifications, presented as advanced oxidation protein products (AOPP) and carbonylated proteins (C=O), in AAA patients qualified for surgery was estimated. The effect of surgical techniques and intraoperative and postoperative treatment on AOPP and C=O levels was evaluated.

**Patients:**

The EVAR group, consisting of 30 patients, was classified for endovascular aneurysm repair, whereas 28 patients were classified for conventional open repair (OR).

**Methods:**

AOPP and C=O were measured using a colorimetric assay kit.

**Results:**

A significantly lower AOPP level obtained 2-4 days after EVAR surgery in comparison with the value found before surgery was noted. In the case of OR postoperative treatment, a tendency of AOPP level to increase was observed. The tendency of C=O to decrease after surgery in the EVAR group was indicated. However, the C=O level tended to increase after OR surgery and reached a significantly higher value 5-7 days after surgery compared with the value obtained before surgery.

**Conclusions:**

Based on our results, it may be concluded that AAA as well as surgical technique contribute to the formation of AOPP and C=O. The analysis of changes in AOPP and C=O values obtained after surgery revealed a significant effect of a patient's condition before surgery as well as the choice of surgery technique on the values of the studied parameters revealed during postoperative treatment.

## 1. Introduction

Recent years have seen a rapid increase in the number of patients with AAA [1, 2]. It has been also estimated that men aged 55-60 suffer from AAA more frequently than women [2–4], whereas the risk of aneurysm significantly increases in females over 70 years of age [5]. Numerous clinical and epidemiological studies point to the complex pathogenesis of AAA and, at the same time, identify proteolytic degradation of aortic wall connective tissue, inflammation and immune responses, and molecular genetics susceptibility as the main mechanisms relevant to AAA formation [6, 7]. Recently the role of oxidative stress in the pathogenesis of AAA has been frequently discussed [8–10].

In the present study we focused on two selected oxidative modifications of proteins (AOPP and C=O) among patients with AAA. AOPP are formed as the result of the reaction between chlorinated oxidants and plasma proteins. This group of modified proteins includes dityrosine, pentosidine, and carbonyl-containing protein products (reactive C=O). In contrast, carbonylation of proteins' markers may be due to oxidation of side chains of amino acids, such as lysine, methionine, and arginine, or through their interaction with reactive oxygen species (ROS) and nonoxidative reactions with carbonyl-containing oxidized lipids [11].

In recent years, a rapid increase in studies focusing on the measurement of oxidatively modified proteins levels in the pathogenesis of various diseases in body fluids or tissues has been observed. On the other hand, several reports have indicated the effect of antioxidants on the oxidative modification of proteins* in vitro*. The incubation of plasma with tert-butylhydroperoxide (t-BHP) resulted in significant increase in AOPP and C=O compared to plasma without initiator of oxidative stress [12]. Furthermore, the addition of resveratrol significantly decreased the level of oxidatively modified proteins, which indicates the protective function of resveratrol in a dose-dependent manner against oxidative stress [12]. Another research interest is to analyze the effect of surgery on the intensity of oxidative stress and level of oxidatively modified biomolecules. Rutkowska and coauthors indicated the influence of critical ischemia on the process of oxidative damage of proteins [13]. Furthermore, they demonstrated that the concentration of C=O in serum of patients with chronic arterial occlusion due to atherosclerosis obliterans reached the highest value before surgery and the tendency to increase in the following days (9-14) of the postoperative treatment.

A specific chemical nature of the studied oxidatively modified protein products contributes to the disturbances in their catabolism and their subsequent accumulation. Furthermore, the accumulation of AOPP and C=O promotes oxidative stress and inflammation, which leads to the progression of atherosclerosis [11]. In this context, it is worthwhile to note that aneurysm formation is associated with the atherosclerotic process in more than 90% of AAA cases which are located below the renal arteries [11]. Despite the fact that surgical techniques are constantly improving, the total mortality rate in AAA constantly approaches 90% (especially in cases of aneurysm rupture) [14]. However, it should be clarified that both progressiveness of the disease and invasiveness of these surgical interventions are burdened with risk.

It is well documented that the reperfusion of ischemic tissue due to cross-clamping of the aorta following AAA surgery generates free radicals (FR) and ROS and, therefore, intensifies oxidative stress [15–17]. In contrast, no cross-clamping as well as a relatively brief period of aortic occlusion during EVAR surgery is associated with a lower induction of oxidative stress [17, 18]. Some studies have indicated that preoperative oxidative stress markers' levels, such as malondialdehyde (MDA), the most frequently analyzed product of oxidative modifications of lipids, are higher in EVAR patients, as compared to OR patients [8, 19].

However, because of the significant differences in the intraoperative procedures involved in EVAR and OR, the evaluation of oxidative stress intensity seems to be clinically interesting. Certainly, the influence of the chosen surgical techniques (choice often dictated by clinical reasons) is important, as well as the study of its impact on the analyzed modifications, measured at specific intervals after surgery. The last issue is poorly investigated, particularly in the period longer than 48 hours after surgery. Therefore, the aim of the study was to evaluate AOPP and C=O in patients scheduled for AAA surgery and the influence of intraoperative and postoperative treatment on AOPP and C=O levels. Finally, the effect of two different surgical techniques (EVAR and OR) on the selected oxidative modifications of proteins was also analyzed.

## 2. Materials and Methods

### 2.1. Study Population

The study protocol conforms to the ethical guidelines of the World Medical Association Declaration of Helsinki. The present study is the continuation of previous work in which number approval decision of the Bioethical Commission, study criteria, exclusion criteria, and clinical examination were described [11]. Preoperative clinical data (regarding cardiovascular risk factors, in particular), diagnosed concomitant diseases, and medications taken by particular patients are listed in Table 1, whereas Table 2 presents the biochemical characteristics of all the patients qualified for the study.

### 2.2. EVAR and OR Patients

The study was performed in a group of 58 AAA patients (45 men and 13 women), who were admitted to the Department of General and Vascular Surgery at the Poznan University of Medical Sciences, Poznań, Poland. Before surgery, Doppler ultrasonography, computed tomography, and arteriography had been carried out. The patients were scheduled for elective surgery, based on the size and growth rate of the aneurysms as well as recommendations of the International Society for Cardiovascular Surgery (> 4 cm in aneurysm diameter or more than twice the diameter of the normal infrarenal and/or expansion rate of ≥ 0.5 cm per 6 months) [19]. Based on the interview and the clinical examination, 30 patients (21 men and 9 women) were classified as a high-surgical-risk and/or with anatomic characteristics favorable to EVAR. In contrast, 28 patients (24 men and 4 women) were classified as low-surgical-risk and eligible for OR. The main criteria for high-surgical- or anesthetic risk patients were characterized in a previous study [20].

The drugs administered before, during surgery and postoperatively were listed in a previous study [20]. Shortly, all the patients were on the statins on the day of surgery. The administration of *β*-blockers and ACE inhibitors was continued until surgery. Moreover, all patients received the following drugs: cetirizine hydrochloride before the surgery, mannitol and hepatin before clamping of the aorta, and antibiotics and nimesulide for 72 hours postoperatively.

### 2.3. Sample Collection

Venous blood samples were drawn preoperatively, before the induction of anesthesia (called “before surgery”), and postoperatively, 1 day after operation (called “1 day after operation”) and 2-4 days after operation (called “2-4 days after operation”), from the arms of patients in the recumbent position. According to longer hospitalization after OR surgery, an additional blood sample after 5-7 days was collected. Furthermore, blood samples were also collected intraoperatively, after the implantation of the vascular prosthesis, 5 min after reperfusion for OR patients, and after the placement of a stent graft, 5 min after reperfusion for EVAR patients (called “reperfusion”). Samples were collected into heparin anticoagulant tubes. After 30 minutes, the tubes were centrifuged at 3.000 rpm for 15 minutes. Plasma samples were stored at the temperature of −80°C until all assays were performed.

### 2.4. Biochemical Parameters

The Medonic M20 automatic analyzer (Clinical Diagnostic Solutions, Inc., USA) was used to determine the following preoperative parameters: white blood cells count (WBC), red blood cells count (RBC), hemoglobin (HGB), hematocrit (HCT), and blood platelets (PLT). Blood biochemical analysis was performed using the EasyRA analyzer (Medica, USA) and included the determination of total cholesterol (TC), LDL-cholesterol (LDL-C), HDL-cholesterol (HDL-C), triacylglycerols (TAG), glucose (G), uric acid (UA), fibrinogen (FB), creatinine (Cr), urea (U), and albumin.

### 2.5. AOPP, Protein Carbonyl Content, and Total Protein Content

AOPP (OxiSelect AOPP Assay Kit; Cell Biolabs, Inc., USA), protein carbonyl content, and total protein content (Protein Carbonyl Colorimetric Assay Kit; Cayman Chemical Company, USA) analyses were performed according to the methods published previously by Gryszczyńska et al. [11]. Shortly, the chloramine reaction initiator was used to measure the concentration of AOPP, which was expressed per total protein content (*μ*mol/mg protein). The concentration of C=O was measured based on the reaction between protein carbonyls and 2,4-dinitrophenylhydrazine (DNPH), whereas dilution of proteins with guanidine hydrochloride followed by measurement of the absorbance at 280 nm was used to determine the total protein content. Colorimetric assay kits were carried out using the Zenyth 200 Microplate Spectrophotometer (Anthos Labtec Instruments GmbH).

### 2.6. Statistical Analysis

The statistical analysis was conducted using GraphPad Prism software 5.0 (GraphPad Software, San Diego, CA). The Kolmogorov-Smirnov or Shapiro-Wilk statistical test was used to analyze the normality of quantitative variables. Any parameter not following the normal distribution was analyzed by the Mann-Whitney test and presented as median and interquartile ranges. Normally distributed, continuous variables were analyzed using Student's *t*-test. The Pearson or the Spearman correlation coefficient was used to test the strength of any associations between different variables. In all cases,* P* value ≤ 0.05 was considered significant.

## 3. Results

**Figure 1:**
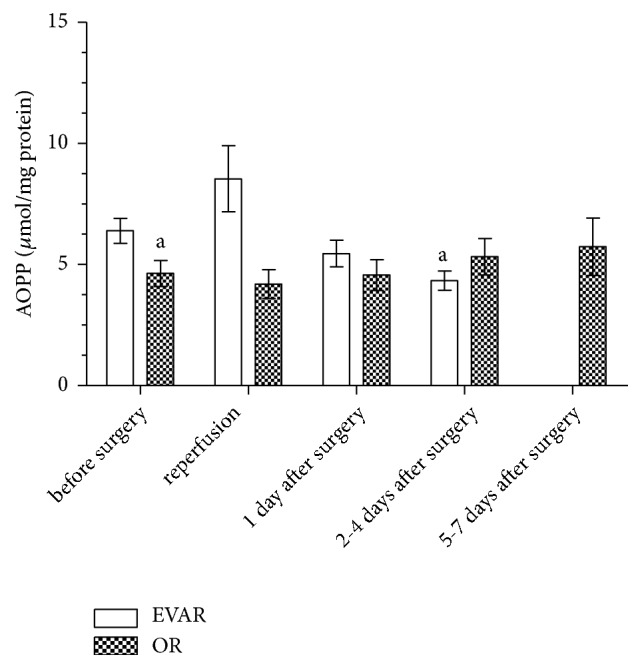
*The concentration of AOPP in the serum of patients qualified for EVAR and OR before surgery, intraoperatively, and during postoperative treatment expressed as  μmol/mg protein*. ^a^Significant difference versus EVAR before surgery.

The results obtained in this study indicate the effect of the type of surgery as well as the type of intraoperative and postoperative treatment on AOPP levels. The plasma concentrations of AOPP in EVAR and OR patients are presented in Figure 1. The average of AOPP levels in EVAR patients was significantly higher (mean (SD), 6.40 (2.70) *μ*mol/mg protein) in comparison with the OR group (mean (SD), 4.63 (2.80) *μ*mol/mg protein) prior to surgery. The investigation of the effects of EVAR surgery and postoperative treatment showed a tendency of the AOPP level to increase after reperfusion and a tendency to decrease 1 day and 2-4 days after surgery. A significant decrease in AOPP concentration after 2-4 days (mean (SD), 4.34 (1.94) *μ*mol/mg protein) of postoperative treatment was observed. In contrast, while analyzing OR postoperative treatment we observed a tendency of the AOPP level to increase (1 day after: median (range), 3.47 (0.68-15.07) *μ*mol/mg protein; 2-4 days after: mean (SD), 5.32 (3.09) *μ*mol/mg protein; 5-7 days after: mean (SD), 5.74 (4.26) *μ*mol/mg protein).

**Figure 2:**
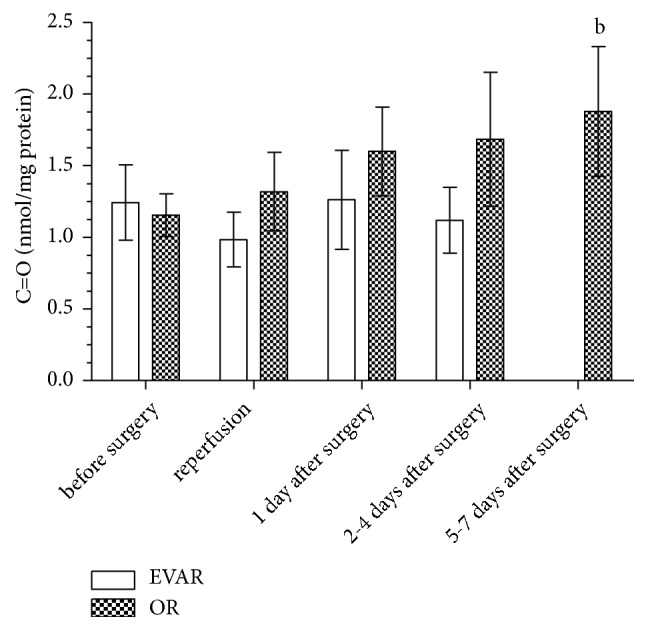
*The concentration of C=O in the serum of patients qualified for EVAR and OR before surgery, intraoperatively, and during postoperative treatment expressed as nmol/mg protein*. ^b^Significant difference versus OR before surgery.

The plasma contents of C=O in EVAR and OR patients are shown in Figure 2. The C=O level was similar in EVAR (median (range), 0.83 (0.22-5.95) nmol/mg protein) and OR patients (median (range), 1.00 (0.20-3.10) nmol/mg protein) before surgery. The average values of C=O concentrations in the EVAR group during postoperative treatment were found not significant in comparison to the value found before surgery. Nevertheless, the analysis of the effects of EVAR surgery and postoperative treatment showed a tendency of the C=O level to decrease after reperfusion and a tendency to decrease 2-4 days after surgery. It is worth noting that the C=O level tends to increase after OR surgery (1 day after: median (range), 0.90 (0.21-6.84) nmol/mg protein; 2-4 days after: median (range), 1.02 (0.44-7.60) nmol/mg protein) and reaches a significantly higher value 5-7 days after surgery in comparison to the values obtained before surgery (5-7 days after: median (range), 1.35 (0.33-6.00) nmol/mg protein).

The changes in AOPP and C=O values in relation to the value found before surgery (Δ) and in intraoperative treatment (reperfusion) (Δ1) were calculated (Table 3). The change in AOPP value after reperfusion was found to be positively correlated with the value found before surgery in the OR group (r=0.51). Furthermore, it was found that the changes in AOPP concentration obtained in postoperative treatment were positively and significantly correlated with the value found before surgery in the EVAR group (1 day after: r=0.57; 2-4 days after: r=0.63) as well as the OR group (2-4 days after: r=0.53). In the EVAR group, a significant correlation between ΔC=O obtained in intraoperative treatment and the level found before surgery was noted. The data collected in Table 3 also shows a positive and significant correlation between ΔC=O found after reperfusion and 1 day as well as 2-4 days after surgery and the C=O level found before surgery in OR patients (reperfusion: r=0.76; 1 day after: 0.58; 2-4 days after: r=0.48). The statistical analysis of the changes in AOPP also showed that Δ1AOPP values obtained 1 day and 2-4 days after surgery were positively correlated with the level found in intraoperative treatment (reperfusion) in EVAR patients (1 day after: r1=0.60; 2-4 days after: r1=0.71). The correlation between changes in AOPP obtained during postoperative treatment and the level found for reperfusion was not found in OR. Moreover, positive and significant correlations were found for Δ1C=O obtained 1 day after EVAR surgery (1 day after: r1=0.73) and 1 day as well as 2-4 days after OR surgery (1 day after: r1=0.45; 2-4 days after: r1=0.72) with the level found for reperfusion.

The results of the present study also indicate significant correlations for oxidatively modified proteins and biochemical parameters in the plasma collected before surgery in the analyzed groups (Table 4). The statistical analysis of the results obtained for EVAR patients showed a positive and significant correlation between AOPP found before surgery and LDL-C concentration as well as for C=O and UA. In the OR group, the AOPP level analyzed before surgery was found significantly correlated with HGB, HCT, and TG. Correlations' coefficients in the analyzed groups are listed in Table 4.

## 4. Discussion

Different factors involved in AAA development, such as atherosclerosis, inflammation, and proteolysis, have been postulated by many researchers [11, 20]. Recent years have seen a rapid increase in studies focusing on the disruptive role of oxidative stress in remodeling of the abdominal aorta in AAA pathogenesis [9, 16]. In our previous research, we demonstrated that vascular disease, AAA, and aortoiliac occlusive disease (AIOD) contribute to the increase formation of AOPP and C=O [11]. In the present study, the classification of AAA patients as high- and low-surgical-risk reveals a significantly higher AOPP level in patients qualified for EVAR repair, compared to OR patients. This relationship does not seem to be surprising, since EVAR is usually recommended for older patients with different comorbidities, and therefore the greater oxidative stress accompanying them certainly affects the modification of proteins.

Literature data shows that surgery exerts its influence on plasma prooxidant-antioxidant balance in AAA patients [21, 22]. An inflammatory response as well as an intensified generation of FR results from the reperfusion of ischemic tissue due to cross-clamping of the aorta following OR [16, 17]. In contrast, in EVAR patients, another research demonstrated lower production of FR and ROS due to lack of intra-abdominal manipulation and a relatively brief period of aortic occlusion [21, 23]. Furthermore, based on the literature data we expected that the FR generated during a brief period of ischemia in EVAR patients could evoke an antioxidant response [23–25]. AOPP values found in intraoperative treatment (reperfusion) in EVAR and OR patients did not confirm this hypothesis. This phenomenon may be explained by the fact that the body's defense system is probably insufficient in EVAR patients, due to various coexisting diseases. It should also be pointed out that anesthetic drugs, such as propofol and sevoflurane, were administered to all the patients to protect tissue against injury during surgery. Some studies demonstrated that the lipid peroxidation inhibitory properties of propofol were reflected in a decreased MDA level [26, 27]. Nevertheless, the mechanism of propofol and sevoflurane, as well as other drugs mentioned in Table 1, against oxidative modification of proteins is yet unknown. Probably, they may not have such properties, which was reflected as a tendency of the AOPP level to increase as compared to AOPP values found before surgery.

For C=O levels after reperfusion, an insignificantly higher level was observed for OR patients as compared to the EVAR group. Based on the present study, it is difficult to clearly interpret the inverse tendency in AOPP and protein carbonylation levels between the studied groups. In our opinion, there are two plausible explanations. The first one assumes that spectrophotometric measurement of AOPP and C=O concentration is not specific [11]. Based on the literature and our previous and present results, we may speculate that spectrophotometric measurement of protein-hydrazone concentration does not reflect all carbonylated proteins [11, 28]. Moreover, DNPH does not react with highly modified proteins or less available C=O groups [11]. Due to the fact that the molecular composition of AOPP has been so far poorly characterized, the chloramine T reagent applied in the AOPP spectrophotometric assay kit is not specific for the particular “subgroup” of AOPP.

Furthermore, based on the study of Majewski and coworkers, who suggested that MDA is a final product of lipid peroxidation derived from polyunsaturated fatty acids and that oxidized low density lipoproteins (ox-LDL) are probably formed earlier, the kinetics of oxidative modification of proteins seem to be worth similar consideration [17]. Therefore, we may suggest that different types of oxidatively modified proteins are formed in different stages of oxidative stress. We may then hypothesize that the increase in AOPP concentration after reperfusion in EVAR patients is the result of rapid oxidation of proteins by chlorinated oxidants. Probably, the carbonylation of proteins takes place at a slower rate. Furthermore, AOPP and C=O may circulate in patients' blood for a longer time or AOPP may be finally converted into C=O. However, further experiments would be required to verify the abovementioned hypothesis. Nevertheless, it can be concluded that the elevated levels of C=O and AOPP reflect the intensity of oxidative stress as well as the impairment of proteins' function. Numerous clinical and epidemiological studies point to the intestine, heart, and lungs as the most sensitive organs to ischemia-reperfusion injury [4]. Furthermore, some studies indicated an intensified systemic inflammatory response, synthesis of acute-phase proteins, hormones, cytokines, and ROS formation after AAA repair [4, 29]. Aivatidi and coworkers noticed that almost all oxidative stress parameters returned to their normal values within 24 hours [18]. Majewski and coworkers demonstrated that FR levels, measured by electron paramagnetic resonance spectroscopy spin-trapping at the beginning of reperfusion, decreased 24 hours after AAA surgery due to various endogenous antioxidants and antioxidant therapies [17]. On the other hand, Lindsay and coworkers demonstrated a significantly elevated F_2_-isoprostane level through the perioperative period and further significant increases on the third and fifth day after surgery in patients with raptured AAA [30]. In the present study, a significantly lower AOPP level 2-4 days after EVAR surgery as compared to its value prior to surgery was observed. EVAR is a minimally invasive technique, characterized by a shorter procedure time, limited number of general anesthesia, and reduction in perioperative injury, postoperative pain, blood loss, and perioperative mortality as compared to OR [31]. The other advantage brought by EVAR, as a result of the abovementioned benefits, is reduced oxidative stress. In this regard, we can conclude that a significant decrease in AOPP concentration found after EVAR surgery is the result of the less invasive surgical technique, reduced oxidative stress, and activation of an antioxidant response during postoperative treatment. While AOPP for EVAR patients decreased during postoperative treatment, it increased insignificantly finally to reach a significantly higher value after 5-7 days for the OR group. In our opinion, a tendency of the AOPP level to increase during postoperative treatment may reflect adverse/destructive effect of conventional open repair on plasma antioxidant-prooxidant balance in AAA patients. Furthermore, we can assume that OR surgery has a long-term implication for AAA patients' antioxidant status.

In order to demonstrate that postoperative levels of oxidatively modified proteins strictly depend on a patient's condition prior to surgery as well as the surgery technique and reperfusion, the changes in values (Δ, Δ_1_) were calculated. The negative ΔAOPP and Δ_1_AOPP values for EVAR patients found during postoperative treatment may reflect the decrease in oxidative modifications of proteins. In contrast, the positive ΔAOPP, Δ_1_AOPP, ΔC=O, and Δ_1_C=O values for OR patients may indicate the inability to inhibit protein modification by the body's antioxidant system. Moreover, positively correlated changes in C=O and AOPP values during postoperative treatment, as opposed to the values found before surgery, and the occurrence of reperfusion may suggest that both a patient's condition prior to surgery and the surgery technique influence the concentration of oxidatively modified proteins. Interestingly, the increase in ΔAOPP found in intraoperative treatment (reperfusion) was observed in the EVAR group. In spite of the lower invasiveness of the EVAR technique, a short procedure time, and a brief period of ischemia in comparison to OR, we can not state unequivocally that endovascular aneurysm repair reduces the oxidative modification of proteins, which may be probably associated with a patient's condition before surgery. On the other hand, intensified generation of FR during brief ischemia of EVAR may evoke an antioxidant response reflected as a tendency of AOPP and C=O levels to decrease in 2-4 days after repair. Interestingly, Rutkowska and coworkers demonstrated a negative correlation between ΔC=O during postoperative treatment and the values found before surgery in serum of men with chronic arterial occlusion, which probably reflects the regulatory effect of the body's antioxidant system [13]. According to our results, we suggest that OR causes longer recovery and indicates the inability of the body's antioxidant system to inhibit protein modification, which is reflected in the highest Δ_1_AOPP and Δ_1_C=O values as well as high C=O and AOPP values found in 5-7 days after surgery. It may be concluded that EVAR patients are characterized by a more effective defense system.

Inflammation is a fundamental factor in the pathogenesis of AAA [32]. Several reports have indicated elevated markers of inflammation in serum of AAA patients in comparison with healthy controls [32–34]. Moreover, the association between serum concentration of inflammatory cytokines and aneurysm diameter was also proved [35]. Tambyraja and coworkers demonstrated a significantly higher level of CRP and higher WBC in patients with a symptomatic intact AAA compared to those with an asymptomatic intact AAA [32]. In the EVAR group, the value of AAA diameter was found significantly correlated with both PLT and WBC levels. It may suggest that AAA diameter and sac volume play a role in the activation of PLT. Furthermore, the relationship between AAA diameter and both PLT and WBC levels may reflect a more intense thrombogenic and inflammatory reaction.

## 5. Study Limitations and Strengths

The main quality of the present study is establishing the effect of OR and EVAR surgery techniques and intraoperative and postoperative treatment on AOPP and C=O levels in AAA patients qualified for surgery. Oxidative modification of proteins is intense during the oxidative stress and easy to detect. However, the choice of markers may be difficult because modifications of amino acids' residues in proteins are varied. In the present study, we used the OxiSelect™ AOPP Assay Kit and Protein Carbonyl Colorimetric Assay Kit, which are simple, rapid, reproducible, and sensitive. One of the most interesting results is a tendency of AOPP and C=O to decrease after surgery in EVAR patients and to increase in the OR group. We may only hypothesize that both types of oxidative modification of proteins take place at different rates. On the other hand, the plasma antioxidant-prooxidant balance in AAA patients is definitely disrupted and the body's defense system is probably not efficient enough, especially in EVAR patients, due to various coexisting diseases. In this connection, despite of the less invasive surgery technique, the tendency of AOPP to increase after reperfusion in EVAR patients was observed. On the other hand, this tendency, which probably reflects generation of free radicals during a brief period of ischemia, could evoke antioxidant system mobilization, for example, via the NRF-2 mechanism cascade (reflected in significant decrease in AOPP after 2-4 days). However, further experiments are required to verify the abovementioned hypothesis.

## 6. Conclusions

It may be concluded that AAA formation contributes to the formation of AOPP and C=O. The classification of AAA patients according to surgical risk revealed a significantly higher AOPP level in EVAR patients, as the result of various coexisting diseases. The analysis of changes in AOPP and C=O values obtained after surgery revealed a significant effect of a patient's condition prior to surgery. Furthermore, positive correlations between Δ_1_AOPP and Δ_1_C=O with values obtained in intraoperative treatment confirmed a significant effect of the surgery technique on values found during postoperative treatment. The comparison between two different surgery techniques showed that open surgical repair is associated with more intensified oxidative stress in postoperative treatment. On the other hand, decrease in AOPP and C=O levels during postoperative treatment of EVAR patients may indicate inhibition of protein modification by the body's antioxidant system.

## Figures and Tables

**Table 1:** Clinical characteristics of the analyzed groups of patients with AAA.

Parameters	EVAR (30 patients)	OR (28 patients)
	No. (%)	No. (%)
Age	67.3±10.8	70.7±7.5
Gender (male/female)	21/9	24/4
Hypertension	24 (80)	18 (28.6)
Hypercholesterolemia	3 (10)	2 (7)
Coronary artery disease	9 (30)	1 (3.5)
Previous myocardial infarction	7 (23.3)	5 (17.9)
Cerebrovascular accident	4 (13.3)	3 (10.7)
Kidney disease	5 (16.7)	3 (10.7)
Pulmonary disease	4 (13.3)	3 (10.7)
*Medications*		
*β*-blocker	15 (50)	14 (50)
ACE	16 (53.3)	15 (53.6)
Statins	15 (50)	14 (50)
NSAIDs	30 (100)	28 (100)

Age presented as mean±SD.

Abbreviations: ACE, an angiotensin-converting-enzyme inhibitor; EVAR, endovascular aneurysm repair; NSAIDs, nonsteroidal anti-inflammatory drugs; OR, open repair.

**Table 2:** Biochemical parameters in EVAR and OR patients' plasma before surgery.

Parameters	EVAR	OR
BMI, kg/m^2^	26.53±5.19	26.83±4.47
TC, mmol/l	4.87±1.21	3.93±0.91^a^
LDL-C, mmol/l	4.35±10.56	2.04±0.88^a^
HDL-C, mmol/l	1.39±0.46	1.21±0.41
TAG, mmol/l	1.63±0.70	1.56±1.03
G, mmol/l	6.20±1.35	6.46±1.18
UA, *μ*mol/l	353.90±123.80	353.90±72.44
WBC, x 10^9^/l	8.90±3.94	7.60±2.46
RBC, x 10^12^/l	4.51±0.54	4.60±0.47
HGB, mmol/l	8.46±1.00	8.55±0.78
HCT, l/l	0.41±0.05	0.41±0.04
PLT, x 10^9^/l	242.1±74.5	243.0±110.6
eGFR, ml/min/1.73 m^2^	69.05±19.27	72.14±15.70
Cr, *μ*mol/l	106.6±58.2	94.8±23.3
FB, mg/dl	338.5±81.4	326.2±108.3
U, mmol/l	6.65±3.58	6.20±1.90
Albumin, g/l	38.1±4.6	37.85±2.86
AAA diameter, mm	58.03±12.80	65.0±9.4^a^

Data presented as mean±SD unless otherwise indicated.

Abbreviations: AAA diameter, abdominal aortic aneurysm diameter; BMI, body mass index; Cr, creatinine; eGFR, estimated glomerular filtration rate; FB, fibrinogen; G, glucose; HCT, hematocrit; HDL-C, high-density lipoprotein cholesterol; HGB, hemoglobin; LDL-cholesterol, low-density lipoprotein cholesterol; PLT, platelets; RBC, red blood cells; TAG, triacylglycerols; TC, total cholesterol; U, urea; UA, uric acid; WBC, white blood cells; others, see Table 1.

^a^ *P*≤0.05 compared with EVAR.

**Table 3:** The changes in concentration of AOPP and C=O in relation to values before surgery.

Parameter	EVAR				
	Day after surgery	Mean±SD	Δ [Δ_1_]	*r [r*_1_ *]*	*P *[*P*_*1*_]
AOPP	Before surgery	6.40±2.68			
	Reperfusion	8.54±4.32	2.14	-0.39	0.211
	1 day after	5.46±2.60	-0.94 [-3.08]	0.57 [0.60]	0.006 [0.042]
	2-4 days after	4.34±1.94	-2.06 [-4.2]	0.63 [0.71]	0.001 [0.020]

C=O	Before surgery	1.24±1.37			
	Reperfusion	0.98±0.48	-0.26	0.46	0.212
	1 day after	1.26±1.70	0.02 [0.28]	0.55 [0.73]	0.006 [0.027]
	2-4 days after	1.12±1.13	-0.12 [0.14]	0.39 [0.35]	0.068 [0.391]

	OR				

AOPP	Before surgery	4.63±2.78			
	Reperfusion	4.19±2.97	-0.44	0.51	0.011
	1 day after	4.57±3.30	-0.06 [0.38]	0.30 [0.22]	0.142 [0.288]
	2-4 days after	5.32±3.08	0.69 [1.13]	0.53 [-0.11]	0.035 [0.684]
	5-7 days after	5.74±4.26	1.11 [1.55]	0.09 [-0.21]	0.748 [0.517]

C=O	Before surgery	1.15±0.77			
	Reperfusion	1.32±1.37	0.17	0.76	<0.0001
	1 day after	1.60±1.60	0.45 [0.28]	0.58 [0.45]	0.002 [0.025]
	2-4 days after	1.68±1.87	0.53 [0.36]	0.48 [0.72]	0.060 [0.002]
	5-7 days after	1.88±1.63	0.73 [0.56]	-0.055 [0.04]	0.862 [0.904]

Abbreviations: Δ, changes in AOPP and C=O values in relation to the value found before surgery; Δ_1_, changes in AOPP and C=O values in relation to the value found for reperfusion; *r*, coefficients of correlation between before surgery and Δ; *r*_1_, coefficients of correlation between reperfusion and Δ_1_; *P*, statistical significance for changes in AOPP and C=O values in relation to the value found before surgery; *P*_*1*_, statistical significance inchanges in AOPP and C=O values in relation to the value found for reperfusion.

**Table 4:** The correlations' coefficients for AOPP and C=O in patients' plasma before surgery.

AOPP			

	*r*	*P*	Group of patients

LDL-C	0.4415	0.021	EVAR
C=O	-0.4267	0.026	OR
HBG	0.4208	0.029	OR
HCT	0.4044	0.036	OR
TG	0.5453	0.003	OR

C=O			

	*r*	*P*	Group of patients

UA	0.4644	0.015	EVAR

Abbreviations: AOPP, advanced oxidation protein products; C=O, carbonylated proteins; others, see Table 1.

## Data Availability

The data used to support the findings of this study are included within the article.
